# Artificial Neural Network Modeling of Glass Transition Temperatures for Some Homopolymers with Saturated Carbon Chain Backbone

**DOI:** 10.3390/polym13234151

**Published:** 2021-11-27

**Authors:** Elena-Luiza Epure, Sîziana Diana Oniciuc, Nicolae Hurduc, Elena Niculina Drăgoi

**Affiliations:** “Cristofor Simionescu” Faculty of Chemical Engineering and Environmental Protection, Gheorghe Asachi Technical University, 73 Prof. Dr. Doc. D. Mangeron Street, 700050 Iasi, Romania; oniciuc.sinziana30@gmail.com (S.D.O.); Nicolae.Hurduc@tuiasi.ro (N.H.)

**Keywords:** glass transition temperature, artificial neural networks, QSPR, homopolymers, Bacterial Foraging Optimization

## Abstract

The glass transition temperature (Tg) is an important decision parameter when synthesizing polymeric compounds or when selecting their applicability domain. In this work, the glass transition temperature of more than 100 homopolymers with saturated backbones was predicted using a neuro-evolutive technique combining Artificial Neural Networks with a modified Bacterial Foraging Optimization Algorithm. In most cases, the selected polymers have a vinyl-type backbone substituted with various groups. A few samples with an oxygen atom in a linear non-vinyl hydrocarbon main chain were also considered. Eight structural, thermophysical, and entanglement properties estimated by the quantitative structure–property relationship (QSPR) method, along with other molecular descriptors reflecting polymer composition, were considered as input data for Artificial Neural Networks. The Tg’s neural model has a 7.30% average absolute error for the training data and 12.89% for the testing one. From the sensitivity analysis, it was found that cohesive energy, from all independent parameters, has the highest influence on the modeled output.

## 1. Introduction

Due to their high specific strength, lightweight, high performance, or easy processability, polymers are often chosen as the best option in different applications. Even though the high number of synthesized polymers is impressive, nowadays there is still continuous competition to create new polymers with enhanced or specific end-use properties [1,2,3,4]. However, the maximum utility potential of these materials was not reached. The determination of the relationship between structure and properties can lead to advances in the field and can help in the endeavor to find materials with specific properties. Nevertheless, in the case of polymers, due to their complexity, this attempt is difficult and time-consuming.

A key parameter of polymers, the glass transition temperature (Tg), could be perceived either as an indicator of amorphous content, different degrees of polymerization, the presence of moisture or as additives in these materials. Still, the Tg is not that easy to be determined neither in an experimental or a theoretical manner, because of the multiple factors that control this temperature. In view of the fact that the transition in a rubbery state occurs alongside a temperature range, assigning a unique value is problematic, especially for the wider domains. The most popular instrumental technique for the investigation of Tg is Differential Scanning Calorimetry (DSC), yet it was observed that this method has its limitations. Computational methods and, in particular, molecular dynamic simulations are well suited to the characterization of the structure and dynamics of the polymers at an atomic level. The solvent, the confinement, the difunctional or trifunctional cross-linkers, and other effects on the glass transition were spotlighted by molecular dynamics simulations (MD) [5,6,7,8]. However, the MD method is time-consuming, needs powerful computational resources, and the results have to be carefully selected, mainly when special cases are encountered, like wide transition region [9]. Under these conditions, another easy-to-implement instrument, suitable for Tg prediction was sought. At this point, an Artificial Neural Network approach (ANN), another kind of computational modeling method, seems to be the best way to achieve the desired tasks, knowing the machine learning flexibility to address and solve problems from the most distinct fields of the real world (medicine, forecasting, defense, economics, entertainment, and so on). The main advantage of ANNs is their ability to determine a non-linear complex model that correlates the output with different input data for complicated systems or processes, without requiring a prior model. Capable to extract subtle information, this modeling method could provide from sensitivity analysis, the variables’ contribution to the output data. A sustainable materials development, an accelerated evaluation, or optimization of products could be thus addressed with machine learning, easily overcoming conventional synthesis and characterizing methods [10,11,12,13]. Examples of compounds for which ANNs were successfully used to predict Tg include multicomponent oxide glasses [14], ionic liquids [15], polystyrenes [16,17,18], organic electroluminescent devices materials [19], polyhydroxyalkanoate homo- and copolymers [20], or aromatic polyamides and polybenzimidazoles [21].

Despite the fact that a large amount of data is found in different sources, a unique model that connects the structure and the Tg of polymers was not developed yet. The aim of this study is to take a step further by building a mathematical model, universally available, for the prediction of the glass transition temperature of different kinds of homopolymers with saturated backbones. In this regard, a neuro-evolutive strategy that combines artificial neural networks with a modified bio-inspired metaheuristic represented by the Bacterial Foraging Optimization was applied. The classical Bacterial Foraging Optimization algorithm will be referred to as BFO, while its modified version proposed in this work will be referred to as mBFO. The motivation for choosing ANN-mBFO in detriment of the established classical strategies relies on the following: (i) its easiness of use compared with the phenomenological software modeling approaches that require extensive knowledge; (ii) the generalization capabilities of ANNs strategies that can extract useful information from data; (iii) the efficiency of BFO to solve complex systems. ANNs are simplified mathematical models that imitate the manner in which the brain functions. Although there are significant differences between ANNs and their biological counterpart (in terms of complexity, learning and adaptive mechanisms and capabilities), ANNs proved to be efficient alternative tools that can be successfully used in various applications [22].

In this work, mBFO acts as an optimizer for the ANN architecture and parameters. This strategy for ANN determination belongs to the neuro-evolutive class. The motivation for this selection is based on the following aspects: (i) a simultaneous architecture and internal parameters optimization can be performed; (ii) the shortcomings of gradient descent training algorithms are overcome; (iii) evolution is another form of adaptation. BFO [23] is a population-based bio-inspired metaheuristic that simulates the foraging behavior of *E. coli* bacterium. In combination with machine learning approaches (such as ANNs and Support Vector Machines), it was successfully applied to solve various problems, for instance, rock mass classification [24], Charcot-Matie-tooth disease [25], identification and classification of plant disease [26,27], compact fractal antenna design [28].

The novelty of this work relies on the identification of a simpler mathematical model for Tg that can efficiently replace the complex phenomenological models and the experimental trials, based on a new version of BFO with improved performance.

## 2. Materials and Methods

### 2.1. Database Construction

The current work focuses on developing a neural model that can predict Tg for homopolymers with a saturated backbone. From scientific literature or platforms, only the homopolymers with reported experimental Tg were considered. In most cases, the selected polymers have a -CH_2_-CR_1_R_2_- carbon chain (where R_1_ = -H, -F, -Cl, -CH_3_ or -CN; R_2_ = -F, -Cl, alkyl, cycloalkyl, -CH=CH_2_, -COOH, -COOR, -C_6_H_6_, substituted benzene, -C(=O)NRR’, -OR, -CN or pyridine). Therefore, the database consists of polymers from different families, such as polyolefins, halogenated polyolefins, polyacrylates, polymethacrylates, polycyanoacrylates, polystyrenes/halogenated polystyrenes, poly(vinyl ethers) and polyacrylamides. The poly(vinyl alcohol), one polyglycol and a few polyoxyalkylenes were also considered in the database. The last homopolymers have an oxygen atom in the backbone. The homopolymers considered for ANN are listed in Table S1 (Supplementary Materials).

The structural information used as input data for the ANNs are represented by the number of atoms of the structural units, the weight of the main chain from the structural units (M CB), the weight of non-hydrogen atoms in the main chain of repeat units (M CB-H), and a series of indices: the number of grafts (linear), the presence of a cyclic structure in the side chain (cyc CL), the aromaticity of the side chain (ar CL), the heteroatom presence in the main or side chain (h CB, h CL). The values of the linear indicator could be 0, 1, and 2, depending on the number of grafts. The other indices, cyc CL, ar CL, h CB, h CL, are Boolean types, having an assigned value of 1 if the condition is met.

Other parameters for ANN were those obtained in an empirical manner with the help of the QSPR method through the Synthia module of the Materials Studio software (v 4.0, Accelrys, San Diego, CA, USA) connectivity index 0χ, connectivity index 1χ, van der Waals volume, molar volume at 1 and 298K, density, cohesive energy, and entanglement molecular weight. This empirical method is a rapid property-prediction technique, avoiding molecular dynamics, a complex and time-consuming method.

The zeroth-order (atomic) connectivity indices ^0^χ and the first-order (bond) connectivity indices 1χ are defined as polymer chain connectivity indices, summing the information over the vertices, respectively the edges of the hydrogen-suppressed graph [29].

Van der Waals volume, molar volume at 298 K or even at 1 K expresses volumetric properties. Being a function of the temperature, the molar volume was calculated for 1K to take into account the effects of the frozen-in packing volume at near absolute zero temperature. The van der Waals volume reports the real space occupied by the atoms, without considering the empty space created by the packing of the polymer chain.

A significant parameter that characterizes the polymers is density that indirectly covers the chain packing imposed by tacticity, the degree of grafting or the grafted chain length. The influence of temperature on the density was switched to the two molar volumes previously mentioned.

As it quantifies the intermolecular forces, cohesive energy was considered as an input for the current ANN. This term comprises some factors like crystallinity, dipole moment, and the presence of hydrogen bonds.

Entanglement molecular weight measures the average molecular weight between the entanglements, which relates to the mobility of the chains. This parameter is especially important for the polymers in the rubbery state. The properties were computed for polymers having Mw = 10,000 a.m.u.

### 2.2. Bacterial Foraging Optimization

The modeling procedure is based on ANN architecture and internal parameter optimization using a modified BFO (mBFO) algorithm.

**Figure 1:**
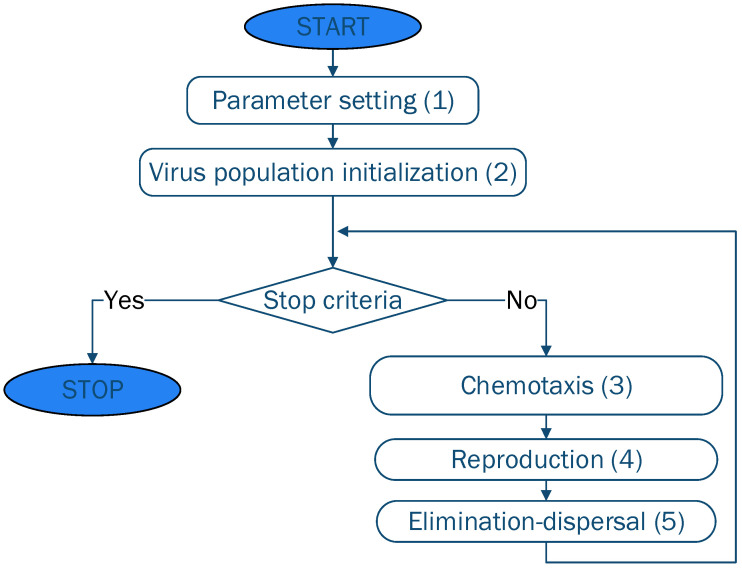
Simplified schema of the BFO algorithm.

In its standard form, as proposed in [23], BFO imitates the concept of the social foraging behavior of bacteria colonies. In the search for food, the bacterium performs small moves in order to maximize the difference between the energy gained from the food and the one burnt. This searching process is referred to as chemotaxis and in the BFO algorithms is simulated through swimming and tumbling using the flagella [30]. Besides chemotaxis, the behavior of *E coli* includes a communication aspect. When stimulated, the bacteria cells release an attractant that allows their aggregation in groups with high density. This process is known as swarming. Moreover, the healthier bacteria undergo asexual reproduction, the cell splitting into two bacteria. This aspect represents the reproduction step. In the final step of the algorithm (elimination and dispersal), some of the cells have a small probability of being randomly eliminated and new replacements are generated. The simplified schema of the BFO algorithm is presented in Figure 1.

The steps indicated in Figure 1 are detailed below:

(1) Parameter setting. Before starting the algorithm, the control parameters of the algorithm are set. BFO has the following parameters that direct the search: population size (S), the life-span of each bacterium controlled in the chemotactic loop (Nc), the swimming length (Ns), the size of the step for the tumble movement (Ci), the number of reproduction loops (Nre), number of elimination-dispersal events (Ned), the probabilities with which the population is subjected to elimination-dispersal events (p_ed_) [30].

(2) Population initialization. Based on the limits of the parameters of the problem being solved, the initial population is generated through the use of random number generators. This strategy is similar to the initialization procedure usually employed by the majority of bio-inspired metaheuristics (Equation (1))

(1) $$x_{i}=\min_{i}+\mathrm{rand}\times \left(\max_{i}-\min_{i}\right)$$

where x_i_ represents the i^th^ parameter of the individual x and min and max represent the minimum and the maximum allowed limits for the i^th^ parameter.

(3) The chemotaxis step focuses on the movement of bacteria based on the perceived concentration of chemical gradients. The movement is composed of two types of rotation: (i) clockwise, which generates a forward movement; and (ii) counter-clockwise, which generates a tumbling movement. The forward movement occurs when a rich gradient is found and the tumbling is performed when a richer gradient is found.

(4) Reproduction focuses on creating copies for 50% of the healthiest individuals. The health of an individual measures its suitability to the environment (how many nutrients have been found and how it was able to avoid noxious substances). This is calculated using a function that in the standard Evolutionary Algorithms terminology is referred to as fitness function.

(5) In the elimination-dispersal step, some of the individuals are replaced with new individuals. This replacement occurs with a p_ed_ probability. Its aim is to refresh the population and to avoid the cases when all individuals are concentrated in a particular area (local minima). In the initial BFO algorithm, p_ed_ is fixed and thus, all individuals have the same replacement probability. Thus, there is the possibility of replacing very good solutions and introducing new worse ones that are far from the optimum. Therefore, in this work, the p_ed_ is modified adaptively, this being one of the specific characteristics of modified BFO (mBFO) (Equation (2)). The aim of this change is to eliminate the issues introduced by the fixed p_ed_ value and to ensure that the good solutions have a lower probability of being replaced.

(2) $$p_{\mathrm{ed}}=\{\begin{array}{c} \frac{{\mathrm{fit}}_{\min}+{\mathrm{fit}}_{\mathrm{avg}}}{{\mathrm{fit}}_{\max}+{\mathrm{fit}}_{\min}},\;\mathrm{if}\;\mathrm{the}\;\mathrm{objective}\;\mathrm{is}\;\mathrm{fitness}\;\mathrm{minimization}\\ \frac{{\mathrm{fit}}_{\max}-{\mathrm{fit}}_{\mathrm{avg}}}{{\mathrm{fit}}_{\max}-{\mathrm{fit}}_{\min}},\;\mathrm{if}\;\mathrm{the}\;\mathrm{objective}\;\mathrm{is}\;\mathrm{fitness}\;\mathrm{maximization} \end{array}$$

where fit_min_ represents the minimum of the fitness of all individuals, fit_max_ the maximum and fit_avg_ the average.

Another modification performed in this work consists of the introduction of an additional step that performs a local search. The objective is to locally improve the so far best solution using a combination of Random Search Algorithm and Opposition Based Learning (OBL) [31]. This idea of locally improving the solutions allows the algorithm to additionally exploit an identified region and improves the probability of finding solutions with high performance [32].

### 2.3. Artificial Neural Networks

The resulting mBFO algorithm is applied to optimize the ANN that models the considered process. The type of ANN used in this work is represented by a Feed Forward Multilayer Perceptron (MLP). MLP is one of the most used types of ANNs [33]. A MLP is formed of perceptrons organized into layers and each layer is fully connected with the next layer. As this type of organization is not compatible with the structure of the mBFO individual (which is a vector of real numbers), a direct encoding is applied to transform the ANN (also known in the neuro-evolutive terminology as the phenotype) into the genotype.

When combining meta-heuristics with ANNs, evolution can be performed at three levels: (i) connection weights (which corresponds to the training phase and is formulated as the minimization of the Mean Squared Error (MSE)—described by Equation (3)); (ii) architecture (which corresponds to the process of identifying the optimal topology); and (iii) learning rules (which aims to adapt the learning rules using the evolutive process) [34]. In the current work, mBFO is applied for a simultaneous optimization of connection weights and architecture. Thus, the fitness function in mBFO is computed based on the MSE determined in the training phase. Moreover, distinctively from other neuro-evolutive strategies, where the activation function is set per layer (which means that all the neurons in the same layer have the same activation function), in the proposed variant, each neuron can have its specific activation function.

(3) $$\mathrm{MSE}=\frac{1}{n}\sum {\left(Y-\;\hat{Y}\right)}^{2}$$

where n represents the total number of exemplars (data) for which the MSE is computed, Y represents the known output, and $\hat{Y}$ is the predicted output.

**Figure 2:**
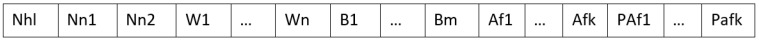
Structure of the genotype representing the evolved ANN parameters.

The complete list of functions a neuron can have is as follows: sigmoid, bipolar sigmoid, logarithmic sigmoid, tangent sigmoid, sinus, radial basis, logistic, tangent hyperbolic, bent identity, soft exponential, linear, leaky relu, and relu. For the functions that need parameters (i.e., sigmoid, bipolar sigmoid, soft exponential), additional memory is allocated. In the end, the genotype (vector of real values), which is the direct encoding of the ANN (phenotype), has the following structure:

In Figure 2, Nhl indicates the number of hidden layers, Nn1 and Nn2 the number of neurons in the first and, respectively, the second hidden layer, W are the weights, B are the biases, Af represents the activation functions, and PAf- the parameters of the activation functions. The limitation to only two hidden layers is imposed based on practical aspects: the higher the number of layers, the higher the number of weights, biases, and activation functions.

### 2.4. Sensitivity Analysis

Sensitivity analysis (SA) for ANNs refers to the determination of the influence of inputs and/or weight perturbations on the model output [35]. SA can be used to determine the importance of model inputs, testing the model conceptualization and improving its structure [36]. There are multiple strategies that can be used to perform SA, among which “clamping” and “Pad” are the most known. Clamping consists of comparing the errors obtained when the original data is applied, with the errors obtained when the inputs of interest are set to a fixed value [37]. In the case of Pad, the sensitivity of each output in relation to a specific input is defined as a differential coefficient. Pad uses partial derivatives and allows to identify the output for small input variations and to perform a classification of the relative contribution of each variant [38]. In this work, the Pad method is used. The exact method of computing the sensitivity is presented in a previous article [39].

## 3. Results

### 3.1. Database

After selecting the published information, the database was constructed with 22 polyolefins, 7 halogenated polyolefins, 13 polystyrene-type compounds, 19 polyacrylates, 29 polymethacrylates, 4 polycyanoacrylates, and 12 homopolymers with hydroxyl or ether groups (Tables S1 and S2). Moreover, six compounds that cannot be included in the previously mentioned classes, the majority of them being polyacrylamides with vinyl backbone are included in the subclass “others”. Where a temperature range was reported, the lowest value was chosen. Moreover, if multiple single values were found, the value with the most bibliographic references was selected.

The dataset regarding QSPR properties selected as input for ANN can be accessed at https://elenadragoi.ro/CV/Documents/BD_ANN_polymers.xlsx, accessed on 23 November 2021. Each one of the inputs has proven its utility, mostly in the case where almost all the parameters are identical. For instance, poly (2-chlorostyrene) and poly (3-chlorostyrene) are different only by the entanglement molecular weight parameter; or poly (3-chlorostyrene) and poly (4-chlorostyrene) are differentiated by their cohesive energy value.

The structural parameters that complete the QSPR ones are important for a beneficial “learning” of the polymers by ANN. The argument that sustains this information is the following: the presence of an aromatic structure in the side chain is indicated by the arCL parameter, and this aromaticity cannot be discovered through zeroth-order (atomic) connectivity indices ^0^χ or the first-order (bond) connectivity indices 1χ.

For the properties calculated by the QSPR method, the following standard deviations were reported (BIOVIA, Materials Studio): van der Waals volume 1.8%, molar volume 2.2–2.5%, density 2.5%, cohesive energy (Fedors) at 298 K 3.9%, entanglement molecular weight 30%.

### 3.2. Artificial Neural Network Modeling

In this work, three modeling situations were considered: (i) Case 1, corresponding to the groups of polyolefins and halogenated polyolefins (with a total of 27 samples); (ii) Case 2, corresponding to the groups of polymethacrylates and polycyanoacrylates (with a total of 32 samples); and (iii) Case 3, that corresponds to the entire dataset and includes all the considered homopolymers (with a total of 104 samples). For all three cases, the same type of ANN model is established (feed-forward multilayer perceptron) using the same settings for the mBFO (number of function evaluations = 5∙10^5^, population size = 20).

Prior to model determination, the available data underwent a processing stage, where it was normalized, randomized, and split. In the normalization phase, a Min-Max approach was used and its role was to transform all data in the (−1, 1) interval to level the influence of the inputs with a higher order of magnitude. The role of randomization and splitting is to ensure that in the training and testing sets, randomly distributed points are assigned. In total, 75% of the data was assigned to the training set and 25% to the testing set. The training set is used in the determination of the ANNs, while the testing set is used to measure the generalization capabilities of the identified models.

Since mBFO has a stochastic nature, for each case, a set of 50 simulations were performed, the statistics for all the models obtained being presented in Table 1, where Architecture indicates the arrangement and the number of neurons in layers in the format inputs: neurons in the first hidden layer: neurons in the second hidden layer: outputs. The difference in the number of input neurons is given by the fact that for Cases 1 and 2, some of the inputs were fixed.

In Table 1, the suitability of the models was determined on the fitness function and the correlation (R) is computed using Equation (4). The higher the value for the fitness function, the better the model performed in the training phase. As it can be observed, for the best models, in the training phase, acceptable MSE values were obtained. However, for the testing phase, for Cases 1 and 2, the correlation is very low, indicating that the identified model had a low generalization capability and was not able in all the cases to model the variation of Tg. These findings were also supported by the average absolute error (AAE computed in the testing phase). AAE was 15.79% in Case 1, 21.39% in Case 2, and 12.89% in Case 3. Thus, although the objective of Cases 1 and 2 was to identify improved models for compounds with specific groups, the results showed that the general model (Case 3) is better in terms of generalization and has a better testing performance.

(4) $$R=\sqrt{R^{2}}=\frac{\sum {\mathrm{difY}}_{i}\sum \hat{{\mathrm{difY}}_{i}}}{\sqrt{\sum {\mathrm{difY}}_{i}^{2}}\sqrt{\sum \hat{{\mathrm{difY}}_{i}^{2}}}}$$

where R^2^ indicates the goodness of fit and difY represent the difference between Y and its mean, diff $\hat{Y}$ represent the difference between $\hat{Y}$ and its mean and Y, $\hat{Y}$ have the same meaning as for Equation (3).

**Figure 3:**
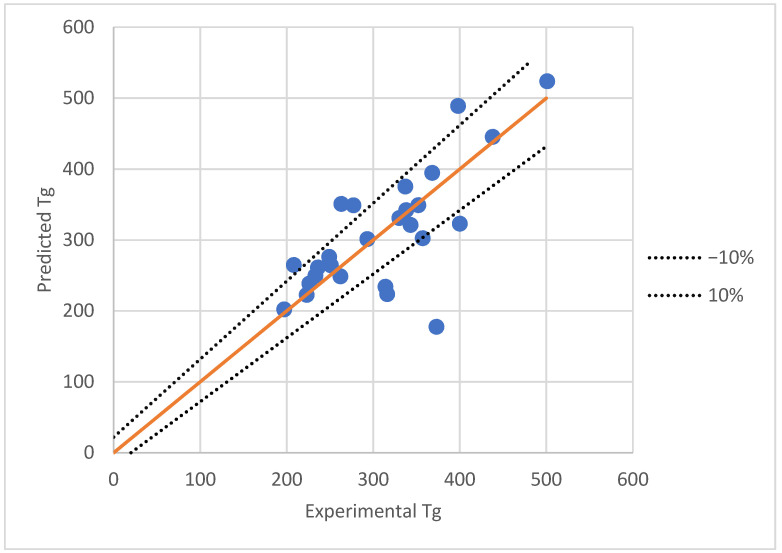
Comparison between experimental and ANN predictions for the testing data in the case of the best model obtained for all the data.

The experimental data versus the predicted values obtained by the best ANN in Case 3 is presented in Figure 3.

As it can be observed, there are a few points where there are significant differences (error > 10%).

The elimination of the three points with the highest error in the AAE determination leads to a value of 8.47%, which is considered acceptable taking into account the high difficulty to reduce a range of temperature to a unique value for Tg.

To validate the model obtained in Case 3, a series of additional samples were collected and used to demonstrate its generalization capability. The AAE obtained for this additional validation set was 15.096% and the correlation was 0.901. These results indicate that the model is able to perform well on unseen data that correspond to the considered criteria and belong to the modeled classes.

In order to determine the influence of each independent parameter on the modeled output, the best model resulting for Case 3 was examined using a sensitivity analysis procedure. The sensitivity values associated with each parameter are presented in Table 2. The higher values of sensitivity indicate a higher influence of the specific parameter in the model.

The mathematical relations describing the best ANN model that can well identify Tg for all the types of compounds considered are presented in the Table S3 (in a Python format) and can be accessed using the following link: https://elenadragoi.ro/CV/Documents/model_from_ANN_polymers.py, accessed on 23 November 2021.

## 4. Discussion

An analysis of the points that do not fit in the interval −10%, +10% from Figure 3 indicate some surprising results, especially since the highest reported value of Tg was chosen for polyacrylonitrile (from class “other”). Poly(chlorotrifluoroethylene), which has the biggest error, has also reported 296 K as Tg data (Aldrich). Still, the deviation remains significant, at 40%. A plausible explication for the high errors of poly(tert-butyl acrylate) and poly(tert-butyl methacrylate), would be that both structures are found in the testing set, and ANN had only one structure with this kind of alkyl group in the training phase, namely the poly (N-tert-butylacrylamide).

In the case of the sensitivity analysis performed, the results are in correlation with the theoretical data. It was stated that cohesive energy is a fundamental property of polymers [29], so the fact that this is the parameter with the highest sensitivity for the neural model is no surprise at all.

The packing regularity can be indirectly found both in the density value and the difference between the molar volume at 298 K and 1 K. However, the density provides information about the degree of grafting or the grafted chain length, so as to understand its favorable position in this ranking. The molar volumes at 298 K and 1 K were thought of as parameters that deliver temperature influence on the polymer behavior. It seems that their dependence and also the van der Waals volume disqualify them regarding their importance for ANN.

Since the entanglement molecular weight is an important parameter only for the polymers in a rubbery state at room temperature, the low sensitivity for the whole series of polymers is understood. The parameters with the lowest influence on the current model are represented by h CB and connectivity index 1χ. These results can then be used further to prune the ANN model.

## 5. Conclusions

The capacity to quantitatively predict the Tg remains a significant obstacle in polymer design. A machine-learning model is a great tool for solving this problem. In the current work, the Tg prediction was performed using a neuro-evolutive strategy combining ANNs and mBFO, a modified version of BFO. The performed modifications in mBFO include the transformation of the p_ed_ fixed variable into an adaptive one based on the fitness function of all individuals in the population and a local search procedure that combines the Local Search algorithm and Opposition Based Learning. The goal of these modifications is to improve the general performance of the algorithm and its capability to efficiently determine good solutions. The role of mBFO was to optimize all the characteristics of the ANNs. A series of structural parameters like the number of atoms, connectivity indices, van der Waals and molar volume, along with cohesive energy and entanglement molecular weight were considered as input for the ANN. The sensitivity analysis revealed that the cohesive energy followed by density and weight of the main chain from structural units significantly affect the Tg values.

Simulations in various cases, focusing on different types of compounds, showed that the mBFO-ANN combination is able to find good models for Tg. However, in terms of testing performance, the best model obtained was the one determined in the case in which all the polymers from the database were taken into consideration, and not for individual groups of polymers. The MSE obtained for the training data was 0.006 and 0.028 for the testing data. The testing AAE was 12.89%, with a few exemplars that had a higher error. When eliminating only three cases, the AAE was reduced to 8.47%. Moreover, an additional set of data was gathered and used for validation. The results obtained indicate that the model was able to provide acceptable predictions even in those cases. Thus, the generalization capability of the identified ANN was demonstrated and the model can be successfully utilized for other compounds from the same classes. Thus, for situations where the Tg is not known and difficult to determine, the model can provide fast predictions based on the known dependent parameters.

## Figures and Tables

**Table 1:** Statistics for the models determined.

Case	Statistic Indicator	Fitness	MSE Training	MSE Testing	Correlation (R) Training	Correlation (R) Testing	Architecture
1	Best	349.9282	0.002858	0.041977	0.976802	0.530663	18:13:01
	Worst	150.3113	0.006653	0.087952	0.944351	−0.15623	18:07:01
	Average	187.8869	0.00552	0.086771	0.954337	−0.05816	
2	Best	1125.606	0.000888	0.039731	0.988932	0.2676	15:08:01
	Worst	213.079	0.00469	0.02958	0.93499	0.12272	15:04:01
	Average	813.29	0.001905	0.033154	0.974491	0.193581	
3	Best	164.8208	0.006067	0.028287	0.920757	0.726176	21:07:01
	Worst	116.8844	0.008555	0.058398	0.88678	0.539156	21:04:01
	Average	132.7765	0.007608	0.049029	0.899501	0.445961	

**Table 2:** Sensitivity analysis for the best model in Case 3.

Order	Input	Sensitivity	Order	Input	Sensitivity
1	Cohesive energy	10.238	12	Molar volume, 1 K	3.668
2	H	9.974	13	Connectivity index ^0^χ	3.479
3	Density	9.314	14	ar CL	3.102
4	N	9.183	15	h CL	3.074
5	M CB	8.023	16	O	2.920
6	Linear	5.185	17	vdW volume	2.386
7	F	5.060	18	Molar volume, 298 K	2.080
8	cyc CL	5.043	19	M CB-H	1.398
9	C	4.836	20	h CB	0.993
10	Cl	4.189	21	Connectivity index ^1^χ	0.951
11	Entanglement molecular weight	3.9295			

## Data Availability

The data presented in this study are available in the Supplementary Materials.

## Supplementary Materials

The following are available online at https://www.mdpi.com/article/10.3390/polym13234151/s1, Table S1: The experimental Tg values of the polymers used as learning and testing data, Table S2: The dataset used for validating the ANN model. Table S3: The ANN model obtained for all homopolymers considered.
