# Supplementary material for: Artificial Neural Network Modeling of Glass Transition Temperatures for Some Homopolymers with Saturated Carbon Chain Backbone
Source: Polymers (Basel). 2021 Nov 27;13(23):4151. doi: 10.3390/polym13234151 (PMC8659568; doi:10.3390/polym13234151)
Supplement: Supplementary file 1 [file polymers-13-04151-s001.zip › polymers-1468432-supplementary.pdf]

__Supplementary Materials__

Artificial Neural Network modeling of glass transition temperatures for some homopolymers with saturated carbon chain backbone

Elena-Luiza Epure*, Sîziana Diana Oniciuc, Nicolae Hurduc and Elena Niculina Drăgoi *

"Gheorghe Asachi" Technical University, Faculty of Chemical Engineering and Environmental Protection, Iasi, 73 Prof. Dr. Doc. D. Mangeron Street, 700050, Romania

* Correspondence: lepure@tuiasi.ro (E-L.E.), elenan.dragoi@gmail.com (E.N.D.);

Table S1. The experimental Tg values of the polymers used as learning and testing data

Index of polymer	Polymer	Tg exp (K)	Reference
**Polyolefins**			
1.	polyethylene	148	[1]
2.	polypropylene	272	[1]
3.	poly(1-butene)	249	[2]
4.	polyisobutylene	209	[2]
5.	poly(4-methyl-1-pentene)	303	[3]
6.	poly(1-hexene)	231	[4]
7.	poly(1-heptene)	228	[5]
8.	poly(1-octene)	208	[5]
9.	poly(1-nonene)	226	[5]
10.	poly(1-decene)	240	[5]
11.	poly(1-undecene)	236	[5]
12.	poly(1-dodecene)	237	[5]
13.	poly(1-hexadecene)	309	[5]
14.	poly(1-octadecene)	328	[2]
15.	poly(vinyl cyclopentane)	343	[5]
16.	poly(vinyl cyclohexane)	400	[5]
17.	poly(1,2-butadiene)	258	[5]
18.	poly(4-cyclohexyl-1-butene)	313	[5]
19.	poly(4-phenyl-1-butene)	283	[5]
20.	poly(3-methyl-1-butene)	323	[5]
21.	poly(5-methyl-1-hexene)	258	[5]
**Halogenated polyolefins**			
1.	poly(chlorotrifluoroethylene)	373	[1]
2.	poly(tetrafluoroethylene)	170	[6]
3.	poly(vinyl chloride)	354	[5]
4.	poly(fluoroethylene)	314	[1]
5.	poly(vinylidene chloride)	255	[1]
6.	poly(1,1-difluoroethylene)	233	[1]
**Polystyrene, Polyhalostyrenes**			
1.	polystyrene	358	[5]
2.	poly(4-fluorostyrene)	368	[2]

3.	poly(2-chlorostyrene)	392	[2]
4.	poly(3-chlorostyrene)	363	[2]
5.	poly(4-chlorostyrene)	383	[2]
6.	poly(2,6-dichlorostyrene)	440	[2]
7.	poly(4-methylstyrene)	370	[2]
8.	poly(2,4-dimethylstyrene)	385	[2]
9.	poly(2,5-dimethylstyrene)	416	[2]
10.	poly(3,5-dimethylstyrene)	377	[2]
11.	poly(4-tert-butylstyrene)	400	[2]

Polyacrylates

1.	poly(acrylic acid)	379	[2]
2.	poly(methyl acrylate)	283	[2]
3.	poly(ethyl acrylate)	249	[2,5]
4.	poly(n-propyl acrylate)	228	[7]
5.	poly(isopropyl acrylate)	262	[2]
6.	poly(n-butyl acrylate)	230	[5]
7.	poly(sec-butyl acrylate)	247	[2]
8.	poly(tert-butyl acrylate)	316	[2]
9.	poly(isobutyl acrylate)	249	[2]
10.	poly(n-hexyl acrylate)	216	[2]
11.	poly(cyclohexyl acrylate)	292	[2]
12.	poly(heptyl acrylate)	213	[7]
13.	poly(dodecyl acrylate)	270	[2]
14.	poli(hexadecyl acrylate)	308	[2,7]
15.	poly(2-ethylhexyl acrylate)	223	[2,7]
16.	poly(benzylacrylate)	279	[2]
17.	poly(2-cyanoethyl acrylate)	277	[2]
18.	poly(2-ethoxyethyl acrylate)	223	[2,7]

Polymethacrylates

1.	poly(methacrylic acid)	501	[2]
2.	poly(benzyl methacrylate)	327	[2]
3.	poly(methyl methacrylate)	378	[5]
4.	poly(ethyl methacrylate)	338	[2]
5.	poly(n-propyl methacrylate)	308	[5]
6.	poly(isopropyl methacrylate	354	[2]
7.	poly(n-butyl methacrylate)	294	[2]
8.	poly(isobutyl methacrylate	326	[2]
9.	poly(sec-butyl methacrylate)	331	[5]
10.	poly(tert-butyl methacrylate)	357	[5]
11.	poly(n-hexyl methacrylate)	268	[2]
12.	poly(cyclohexyl methacrylate)	365	[2]
13.	poly(octyl methacrylate)	203	[5]
14.	poly((dodecyl methacrylate)	208	[2,7]
15.	poly(hexadecyl methacrylate)	288	[2]
16.	poly(octadecyl methacrylate)	173	[2]
17.	poly(2-hydroxyethyl methacrylate)	330	[2]
18.	poly(2-hydroxypropyl methacrylate)	349	[2]
19.	poly(2-phenylethyl methacrylate)	300	[5]
20.	poly(phenyl methacrylate)	383	[2]
21.	poly(4-tert-butylcyclohexyl methacrylate)	371	[8]
22.	poly(2,2,3,3-tetrafluoropropyl methacrylate)	337	[5]
23.	poly(2-tert-butylaminoethyl methacrylate)	306	[2]

24.	poly(2-(diethylamino)ethyl methacrylate)	293	[2]
25.	poly(2-ethylhexyl methacrylate)	263	[2,7]
26.	poly(dimethylaminoethyl methacrylate)	292	[2]
27.	poly(2-ethylbutyl methacrylate)	284	[7]
28.	poly(3,3,5-trimethylcyclohexylmethacrylate)	352	[7]
**Polycyanoacrylates**			
1.	poly(methyl cyanoacrylate)	369	[5,9]
2.	poly(ethyl cyanoacrylate)	406	[5]
3.	poly(isobutyl cyanoacrylate)	403	[5,10]
4.	poly(2-octyl cyanoacrylate)	283	[10]
**Polymers with hydroxyl groups. Polyethers**			
1.	poly (oxymethylene)	191	[5]
2.	poly(ethylene glycol)	213	[11]
3.	poly(epichlorohydrin)	251	[2]
4.	poly(ethyl vinyl ether)	233	[2]
5.	poly(tetrahydrofuran)	189	[2]
6.	poly(trimethylene oxide)	197	[12]
7.	poly(propylene oxide)	198	[2]
8.	poly (cyclohexyl vinyl ether)	354	[2]
9.	poly(vinyl alcohol)	358	[5]
10.	poly(4-vinylphenol)	428	[2]
**Others polymers**			
1.	poly(2-vinylpyridine)	357	[5]
2.	poly(N-isopropylacrylamide)	358	[2]
3.	poly(N,N-dimethylacrylamide)	362	[2]
4.	polyacrylamide	438	[2]
5.	poly(N-tert-butylacrylamide)	415	[13]
6.	polyacrylonitrile	398	[2]

Table S2. The dataset used for validating the ANN model.

Nr.crt.	Polymer	Family	Tg exp (K)	Reference
1.	poly(1-pentene)	polyolefins	221	[14]
2.	poly(perfluoropropylene)	halogenated polyolefins	284	[15]
3.	poly(o-methylstyrene)	polystyrene	388	[15]
4.	poly(α-methylstyrene)	polystyrene	448	[15]
5.	poly(n-nonyl acrylate)	polyacrylates	184	[14]
6.	poly(n-decyl methacrylate)	polymethacrylates	203	[14]
7.	poly(vinyl butyl ether)	polyethers	223	[15]
8.	poly(vinyl propyl ether)	polyethers	246	[16]

Table S3. The ANN model obtained for all homopolymers considered.

INP_0= - 1E-20+0.001 +(CarbonAtoms - 1)*(0.999-0.001) /(22 -1)	(1)
INP_1= - 1E-20+0.001 +(HidrogenAtoms - 0)*(0.999-0.001) /(42 -0)	(2)
INP_2= - 1E-20+0.001 +(OxygenAtoms - 0)*(0.999-0.001) /(3 -0)	(3)
INP_3= - 1E-20+0.001 +(NitrogenAtoms - 0)*(0.999-0.001) /(1 -0)	(4)
INP_4= - 1E-20+0.001 +(FlourAroms - 0)*(0.999-0.001) /(4 -0)	(5)
INP_5= - 1E-20+0.001 +(ChlorAtoms - 0)*(0.999-0.001) /(2 -0)	(6)
INP_6= - 1E-20+0.001 +(MCB - 26)*(0.999-0.001) /(116.5 -26)	(7)
INP_7= - 1E-20+0.001 +(MCB-H - 8)*(0.999-0.001) /(116.5 -8)	(8)
INP_8= - 1E-20+0.001 +(liniar - 0)*(0.999-0.001) /(2 -0)	(9)
INP_9= - 1E-20+0.001 +(cicCL - 0)*(0.999-0.001) /(1 -0)	(10)
INP_10= - 1E-20+0.001 +(arCL - 0)*(0.999-0.001) /(1 -0)	(11)
INP_11= - 1E-20+0.001 +(hCB - 0)*(0.999-0.001) /(1 -0)	(12)
INP_12= - 1E-20+0.001 +(hCL - 0)*(0.999-0.001) /(1 -0)	(13)
INP_13= - 1E-20+0.001 +(0xConectivity- 1.41)*(0.999-0.001) /(17.51 -1.41)	(14)
INP_14= - 1E-20+0.001 +(1xConectivity - 1)*(0.999-0.001) /(23.65 -1)	(15)
INP_15= - 1E-20+0.001 +(vdW - 4.75)*(0.999-0.001) /(228.95 -4.75)	(16)
INP_16= - 1E-20+0.001 +(1KMolarVolume - 19.22)*(0.999-0.001) /(297.72 -19.22)	(17)
INP_17= - 1E-20+0.001 +(298KMolarVolume - 22.99)*(0.999-0.001) /(361.67 -22.99)	(18)
INP_18= - 1E-20+0.001 +(density - 0.85)*(0.999-0.001) /(2.05 -0.85)	(19)
INP_19= - 1E-20+0.001 +(CohesiveEnergy - 8.089)*(0.999-0.001) /(119.098 -8.089)	(20)
INP_20= - 1E-20+0.001 +(Entanglement - 1926)*(0.999-0.001) /(134132 -1926)	(21)
H1_0=math.sin(+INP_0*0.544118033964117+INP_1*0.296772575159206+INP_2*-0.461118930806975+INP_3*-0.80923201982167+INP_4*0.867751477630028+INP_5*0.772613075831481+INP_6*0.624272423518401+INP_7*-0.256325474010821+INP_8*-0.309944358465243+INP_9*-0.0523404383832098+INP_10*-0.79279679144531+INP_11*-0.90163550204181+INP_12*0.186024552652494+INP_13*-0.453917196644759+INP_14*-0.00835685757034888+INP_15*0.0444918686935694+INP_16*0.503578845034187+INP_17*-0.384477748197456+INP_18*-0.741268655224187+INP_19*-0.710252762299149+INP_20*-0.678021701612108+-0.348272897544441)	(22)
H1_1=((math.sqrt(math.pow(+INP_0*-0.0858506360166572+INP_1*0.984645446398686+INP_2*0.226241001048936+INP_3*0.592189048208227+INP_4*0.315858685205579+INP_5*-0.264796635800749+INP_6*-0.0641376465815047+INP_7*0.507587883242079+INP_8*-0.0458796039048231+INP_9*-0.945010728802051+INP_10*0.796790954019358+INP_11*0.300109760761138+INP_12*-0.263023469335923+INP_13*0.539173549148303+INP_14*-0.252898642903707+INP_15*0.215614323985436+INP_16*0.108791471901005+INP_17*-0.575766678803576+INP_18*-0.335848967425074+INP_19*0.0220135960165123+INP_20*-0.438561000238494+0.876196773193087, 2) + 1.0) - 1.0) / 2.0 + INP_0*-0.0858506360166572+INP_1*0.984645446398686+INP_2*0.226241001048936+INP_3*0.592189048208227+INP_4*0.315858685205579+INP_5*-0.264796635800749+INP_6*-0.0641376465815047+INP_7*0.507587883242079+INP_8*-0.0458796039048231+INP_9*-0.945010728802051+INP_10*0.796790954019358+INP_11*0.300109760761138+INP_12*-0.263023469335923+INP_13*0.539173549148303+INP_14*-0.252898642903707+INP_15*0.215614323985436+INP_16*0.108791471901005+INP_17*-0.575766678803576+INP_18*-0.335848967425074+INP_19*0.0220135960165123+INP_20*-	(23)

0.438561000238494+0.876196773193087)	
H1_2=max(0, +INP_0*-0.0617466229099669+INP_1*-0.513938309299112+INP_2*0.852930598164218+INP_3*0.320097301167876+INP_4*-0.188259803749279+INP_5*-0.565019995860616+INP_6*0.595413011965646+INP_7*-0.528003714700397+INP_8*0.300099967291629+INP_9*0.3269811415883+INP_10*-0.518486845145325+INP_11*0.511345684282431+INP_12*0.0173126291726122+INP_13*0.252502578031333+INP_14*0.763151914397945+INP_15*-0.740341906289209+INP_16*0.733733450016088+INP_17*-0.390569334794352+INP_18*0.326451021168223+INP_19*0.0512314716264531+INP_20*0.970935988012847+-0.379816127497597)	(24)
H1_3=max(0, +INP_0*0.251325131076016+INP_1*0.733252093823446+INP_2*-0.914273967786097+INP_3*0.0706894099604163+INP_4*-0.643131686700084+INP_5*0.648492956530627+INP_6*0.313849027566571+INP_7*0.512316739415225+INP_8*0.309558430344284+INP_9*0.56037521974103+INP_10*-0.135646917156795+INP_11*-0.471224746725786+INP_12*0.00402898241176353+INP_13*-0.765585265017006+INP_14*0.102439603569315+INP_15*-0.877832990710656+INP_16*-0.992166160404062+INP_17*0.914592215455382+INP_18*-0.620233399468425+INP_19*-0.45788393027627+INP_20*0.702962238948209+0.235371625639954)	(25)
H1_4=math.exp(-math.pow(-(+INP_0*0.259811423681874+INP_1*-0.34849261239678+INP_2*-0.621987388597616+INP_3*-0.114861640279794+INP_4*0.846994671405101+INP_5*-0.862629829327588+INP_6*-0.481480224445337+INP_7*-0.408832484298989+INP_8*0.431302793050971+INP_9*0.511664918385287+INP_10*-0.490669801410196+INP_11*-0.0156375070074777+INP_12*0.421550034123568+INP_13*-0.132391384285057+INP_14*-0.104625200662426+INP_15*-0.670773667632673+INP_16*-0.427121143765862+INP_17*0.302159948768932+INP_18*0.269347757078597+INP_19*-0.295943606400851+INP_20*0.149878613016874+-0.23607923464563), 2.0))	(26)
H1_5=if((+INP_0*-0.257142883471411+INP_1*-0.95883667915189+INP_2*0.26637878979456+INP_3*0.849509683456387+INP_4*0.134652736795476+INP_5*-0.445959419988526+INP_6*-0.733867809392854+INP_7*0.694400212738213+INP_8*0.564565808158011+INP_9*-0.901316483725244+INP_10*0.527228429936051+INP_11*-0.631443262118635+INP_12*-0.160654672207758+INP_13*-0.0641057318280757+INP_14*0.570546973372971+INP_15*-0.3741120021697+INP_16*-0.00849712043509802+INP_17*-0.222949439894773+INP_18*0.77170636410642+INP_19*-0.12424419376721+INP_20*-0.403498397922002+0.319327922334706)<0): 0.01*(+INP_0*-0.257142883471411+INP_1*-0.95883667915189+INP_2*0.26637878979456+INP_3*0.849509683456387+INP_4*0.134652736795476+INP_5*-0.445959419988526+INP_6*-0.733867809392854+INP_7*0.694400212738213+INP_8*0.564565808158011+INP_9*-0.901316483725244+INP_10*0.527228429936051+INP_11*-0.631443262118635+INP_12*-0.160654672207758+INP_13*-0.0641057318280757+INP_14*0.570546973372971+INP_15*-0.3741120021697+INP_16*-0.00849712043509802+INP_17*-0.222949439894773+INP_18*0.77170636410642+INP_19*-0.12424419376721+INP_20*-0.403498397922002+0.319327922334706)<br>else: (+INP_0*-0.257142883471411+INP_1*-0.95883667915189+INP_2*0.26637878979456+INP_3*0.849509683456387+INP_4*0.134652736795476+INP_5*-0.445959419988526+INP_6*-0.733867809392854+INP_7*0.694400212738213+INP_8*0.564565808158011+INP_9*-0.901316483725244+INP_10*0.527228429936051+INP_11*-0.631443262118635+INP_12*-0.160654672207758+INP_13*-0.0641057318280757+INP_14*0.570546973372971+INP_15*-0.3741120021697+INP_16*-0.00849712043509802+INP_17*-	(27)

0.222949439894773+INP_18*0.77170636410642+INP_19*-0.12424419376721+INP_20*-0.403498397922002+0.319327922334706)	
H1_6=(2.0/(1.0+math.exp(-2 *(+INP_0*-0.167845999124169+INP_1*0.510510063134233+INP_2*0.887363660199696+INP_3*0.273732286001817+INP_4*-0.0964331582734111+INP_5*-0.42633015523921+INP_6*0.0637683883420247+INP_7*0.719825736223102+INP_8*-0.76619521781668+INP_9*-0.489679817692855+INP_10*-0.0409930345792713+INP_11*-0.611323020156219+INP_12*0.824030260922289+INP_13*-0.609655145559236+INP_14*-0.727311891631199+INP_15*-0.464382144117735+INP_16*-0.160804892538074+INP_17*-0.763602928439615+INP_18*0.794358150535703+INP_19*-0.748882843522789+INP_20*-0.429709120008987+0.128538574482799))) - 1)	(28)
OUTPUT_1=math.abs(+H1_0*0.69624012661736+H1_1*0.351211305488293+H1_2*0.447268804289353+H1_3*0.433966914489984+H1_4*0.232405907575948+H1_5*-0.676654671979226+H1_6*0.326955002331831+-0.431485658475907)	(29)
OUTPUT_DENORM1=(OUTPUT_1+1E-20-0.001)*(501-148)/ (0.999-0.001) +148	(30)

References

1. Fox, R.B. Glass transition temperature for selected polymers. *CRC Handbook of Chemistry and Physics* **2017**, *87*, 2006-2007.
2. sigmaaldrich. Availabe online: https://www.sigmaaldrich.com/ (accessed on 10.30.2021).
3. Markova, S.Y.; Gries, T.; Teplyakov, V.V. Poly (4-methyl-1-pentene) as a semicrystalline polymeric matrix for gas separating membranes. *Journal of Membrane Science* **2020**, *598*, 117754.
4. Nouri-Ahangarani, F.; Bahri-Laleh, N.; Nekoomanesh-Haghighi, M.; Karbalaie, M. Synthesis of highly isotactic poly 1-hexene using Fe-doped Mg (OEt) 2/TiCl4/ED Ziegler–Natta catalytic system. *Designed Monomers and Polymers* **2016**, *19*, 394-405.
5. Ellis, B.; Smith, R. *Polymers: a property database*; CRC press: 2008.
6. Calleja, G.; Jourdan, A.; Ameduri, B.; Habas, J.-P. Where is the glass transition temperature of poly (tetrafluoroethylene)? A new approach by dynamic rheometry and mechanical tests. *European polymer journal* **2013**, *49*, 2214-2222.
7. Koleske, J.V. Paint and coating testing manual: of the Gardner-Sward handbook.
8. Gargallo, L.; Russo, M. Effect of structure on glass transition temperatures of poly (methacrylic ester) s with bulky side groups. *Die Makromolekulare Chemie: Macromolecular Chemistry and Physics* **1975**, *176*, 2735-2744.
9. Cheung, K.H.; Guthrie, J.; Otterburn, M.S.; Rooney, J.M. The dynamic mechanical properties of poly (alkyl 2-cyanoacrylates). *Die Makromolekulare Chemie: Macromolecular Chemistry and Physics* **1987**, *188*, 3041-3046.
10. Duffy, C.; Zetterlund, P.B.; Aldabbagh, F. Radical polymerization of alkyl 2-cyanoacrylates. *Molecules* **2018**, *23*, 465.
11. Törmälä, P. Determination of glass transition temperature of poly (ethylene glycol) by spin probe technique. *European Polymer Journal* **1974**, *10*, 519-521.
12. Mark, J.; Chiu, D. Dipole moments of poly (trimethylene oxide) chains. *The Journal of Chemical Physics* **1977**, *66*, 1901-1904.
13. Garay, M.T.; Ruiz, L.; Marín, J.R.; Laza, J.M.; Rodriguez, M.; León, L.M. Associative and segregative phase separations of poly (N-tert-butylacrylamide)/poly (acrylic acid) mixtures. Effect of solvent. *Colloid and Polymer Science* **2010**, *288*, 1593-1599.
14 van Krevelen; D.W., te Nijenhuis, K. Properties of Polymers: Their Correlation with Chemical Structure; Their Numerical Estimation and Prediction from Additive Group Contributions, 4th ed., Elsevier, Amsterdam, The Netherlands, 2009; p.143
15. Shen, M.C.; Eisenberg, A. Glass transitions in polymers, *Progress in Solid State Chemistry*,1967,*3*, 407-481

16 Tighe, B.J.; Mann, A. Adhesive and interfacial phenomena in wound healing, *Advanced wound repair therapies*, Farrar, D.Woodhead publishing Limited, Cambridge, UK, 2011; p 262
